# Improvement of Energy Efficiency and Effectiveness of Cooking for Parabolic‐Type Solar Cooker Used with Activated‐Carbon‐Coated Aluminium Cooking Pot

**DOI:** 10.1002/gch2.201900047

**Published:** 2019-08-07

**Authors:** Anik Goswami, Subhajit Basu, Pradip Kumar Sadhu

**Affiliations:** ^1^ Department of Electrical Engineering Indian Institute of Technology (ISM) Dhanbad Jharkhand 826004 India

**Keywords:** coated utensil, energy, exergy, solar cooker

## Abstract

In India, due to the high price of cooking gases, people still cook with noncommercial fuels like kerosene and firewood. These cause a lot of health problems and also harm the environment. A clean solution to this problem is to use a solar cooker. A solar cooker suffers from the problem of low utility and hence takes a longer period of time for cooking. To counter this problem and reduce cooking time, the solar cooker is tested with charcoal coated utensil. In this paper, a parabolic‐type domestic solar cooker is tested for the same utensil with and without cost effective thermal coating. It is observed that the effectiveness and efficiency of cooking is considerably increased by using the charcoal coating on the utensil. Considering the economic condition of the people of the selected area, this method provides a cost effective way to increase the cooking effectiveness of the solar cooker and reduce dependency on noncommercial fuels.

## Graphical abstract

**In this paper, an activated carbon‐coated aluminium pot** is used as a cooking utensil for solar cookers. Using this utensil, the effectiveness and efficiency of cooking employing the solar cooker is considerably increased. The energy efficiency of the coated utensil is 1.59% higher than that of the normal utensil. This method provides a clean, fast, and economical alternative to traditional cooking systems.

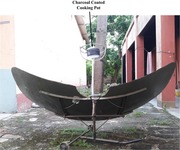

## 1 Introduction

As solar cooker uses solar energy for its operation and provides an effective solution for the energy crisis, which is a major problem.^1^, ^2^ Besides being safe it is also environmentally sustainable as there is no carbon emission.^3^ Fossil fuel has been the main backbone of energy generation in India. However fossil fuel being a nonrenewable source of energy is depleting as a rapid rate. This has led to the increased price of fuel in international market.^4^, ^5^ This has directly affected the price of energy production, resulting in the increase of the price of energy. Considering the economic condition of the region under study, cooking is generally done using noncommercial fuels like kerosene, dung‐cakes, firewood. This fuels have a major problem of causing adverse effects on human health and causes diseases like asthma, pneumonia, and allergy.^6^, ^7^, ^8^ Apart from this these fuels produces a lot of carbon dioxide which contributes to global warming. There is a need to find out alternative cooking medium that is both clean and cheap. India being located in the tropical region, receives a substantial amount of sun light around the year.^9^ The solar energy can be used for cooking using solar cookers which are both simple, easy to operate, and cost effective. Solar cooker suffer from a drawback that the cooking is substantially longer than compared to traditional cooking methods. In order of the utility of solar cooker in India it is ranked at 5th position behind kerosene stove.^10^, ^11^ There is a need to increase the utility of solar cookers in India. To increase the utility of the solar cookers some coating of acetamide and stearic acid is done.^12^, ^13^ Though this method increases the efficiency of the cooker, but there is an increased cost associated with this. Considering the economic situation of the region this method is not a viable solution. Heat exchangers along with the cooker can be used but this too increases the cost of the cooker.^14^ Considering these problems a cost effective way of cooking using existing solar cooker is proposed in this paper. To increase the utility of the solar cooker a lot of advancements is carried out on the cooker. In this paper the traditional solar cooker is used, to increase the utility of the cooking a novel method is proposed utilizing the cooking pot. Most of the household uses aluminium pot for cooking as aluminium utensils is cheaper. Cooking was done using normal aluminium pot and then using aluminium pot coated with charcoal and the energy and exergy analysis was done to determine the efficiency of cooking. In India as primitive methods of cooking using firewood is done a lot of charcoal is available in every house hold. The experiment is performed at Howrah (22°N and 88°E), it lies in the tropics and receives 2528 sunshine hours annually. The mean monthly sunshine hours in October are 182.6. The yearly average solar intensity in Howrah is 4.12 and 4.17 kW m^−2^ day^−1^ in the month of October. Considering the economic status of the said area, solar cooker provides a cheaper alternative as it has no running cost and no fuel cost.^15^, ^16^

## 2 System Description

**Figure 1:**
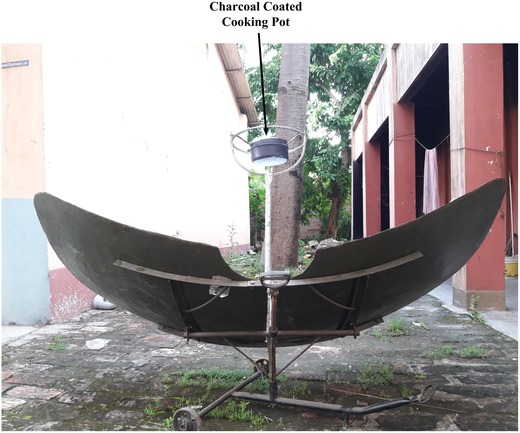
Description of the solar cooker.

The experiment is performed on the roof top of Ramakrishna Mission Shilpamandira (22.64°N and 88°E) in the month of October when the solar intensity remains the highest. This type of solar cooker can produce sufficient heat for cooking. Parabolic cookers can cook faster at high temperature but requires frequent adjustment and supervision for safe operation.^17^ This type of solar cooker is useful for large scale cooking. Basically two aluminium foils are placed parabolically, these two fins reflect and concentrate the sunlight on the cooking top. The bottom of the pot is provided with a heat conducting medium and remaining sides of the pot are blackened. The pot is kept at the center of the focus of two parabolic fins.^18^, ^19^ The capacity of the cooker is 5–10 L. The reflector surface is made up of high reflective chromium nickel sheet. The overall weight is 23 kg. Pot diameter is 300 mm (max). Output power is 600 W at 5 kWh m^−2^ solar insolation. The minimum area required for installation is 2.5 m × 2.5 m. There are two reflector wings each having dimension 0.914 m × 0.914 m. The cooking top is 0.71 m above the base axis as shown in **Figure** **1**. The diameter of the cooking top is 0.305 m. The structural frame is 2.93 m × 0.91 m. The cooking pot has diameter 0.15 m and is placed at the focus of the cooker and is made up of 0.05 m thick galvanized steel. The concentration ratio of the solar cooker reflector is 74.78%. The thin plate type parabolic solar collector can rotate in two axes. To track the Azimuthal angle the solar collector can rotate between 0° to 90°. The collector can rotate from 22° to 66° to track the Zenith angle. By adjusting these angles the maximum intensity of the Sun can be tracked daily and annually. The parabolic type solar cooker is used to boil 1 kg of water. At first the water is boiled using normal aluminium pot and the energy and exergy analysis is performed. The same pot was then coated with charcoal and then the exergy and energy analysis is performed. A comparative study of the energy efficiency is done using the collected data. By using the two different pots the analysis of the efficiency of the solar cooker is presented and the effectiveness of charcoal in increasing the efficiency and decreasing the cooking time is established. The descriptive model set up is shown in Figure 1.

**Figure 2:**
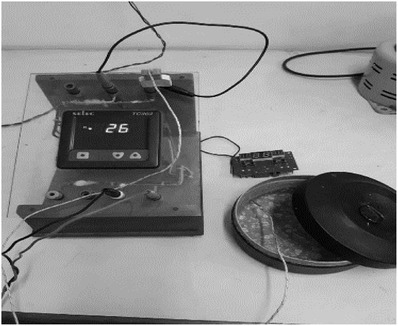
The experimental setup of the measuring system.

The solar cooker is placed in an open area with sufficient irradiation. The maximum insolation is received from 10:00 a.m. to 15:00 p.m.. The experiment is conducted at 12:00 p.m. Water is heated from ambient temperature, after each 3 min the water temperature and solar illumination are being recorded. The total duration of the experiment is 60 min. The experimental setup is shown in Figure 1 and the measurement system is shown in **Figure** **2**. Results are plotted to understand the dependency of heating on solar irradiation and the efficiency of the cooker. Energy and exergy efficiencies provide a tool to analyze the performance of solar cooker. There is a need to get the maximum energy output from the solar cooker so that the efficiency of cooking is improved. Which in turn will decrease the cooking time and provide faster cooking.

## 3 Measurement and Evaluation

The maximum heat energy that can be obtained for any given input is called exergy.^20^, ^21^ It depicts the actual performance of the solar cooker. In exergy analysis the exergy at steps having larger losses are analyzed. The input energy transfer to the output exergy transfer is exergy efficiency.^22^, ^23^

Evaluation of solar energy in the form of input energy of the solar collector is represented as

(1) $$\mathrm{Solar}\;\;\mathrm{radiation}\;\;\mathrm{energy}\;\;(\mathrm{exergy})\;\;=\;\;I_{\mathrm{rr}}\left[1\;\;-\;\;\frac{T_{\mathrm{amb}}}{T_{s}}\right]$$

where *I*_rr_ is solar irradiance intensity, *T*_amb_ is the ambient temperature, and *T*_s_ is the temperature of the Sun which is 5600 K.

The solar cooker output exergy is given as

(2) $$F_{\mathrm{op}}\;\;=\;\;mc_{p}\left(T_{\mathrm{ft}}\;\;-\;\;T_{\mathrm{it}}\;\;-\;\;T_{\mathrm{amb}}\;\ln\;\frac{T_{ft}}{T_{\mathrm{jt}}}\right)$$

The output energy of the solar cooker can be defined from the efficiency of the solar collector that converts the solar radiation energy into heat energy. This heat energy is transmitted to the heating pot. The solar output energy can be defined as

(3) $$E_{\mathrm{op}}\;\;=\;\;mc_{p}(T_{\mathrm{ft}}\;\;-\;\;T_{\mathrm{it}})$$

where *E*_op_ is the output energy, *m* is the mass of the water, *c*_p_ = 4200 J kg^−1^ K^−1^, *T*_ft_ is the final temperature of water, and *T*_it_ is the initial temperature of water.

The intensity of solar radiation is reduced while heating as the temperature of the heating material is very low compared to the temperature of the sun. This causes a difference between the energy and exergy efficiency and is given as

(4) $$e\;\;=\;\;1\;\;-\;\;\frac{T_{0}}{\Delta T}\;\;\ln\left(1\;\;+\;\;\frac{\Delta T}{T_{\mathrm{Wi}}}\right)$$

As the solar cooker fins are constructed from nickel sheet having high reflectivity the effective insolation on the surface of the fins are calculated as

(5) $$I\;\;=\;\;2.16\;\;\times \;\;10^{-5}A_{1}\varepsilon \sigma T_{s}^{4}$$

where 2.16 × 10^−5^ is the solid angle of the sun, ε is the emissivity, and σ is the Stephan–Boltzmann constant.

Considering the reflectivity of the solar cooker as ρ, the emissivity is given as

(6) $$\varepsilon_{1}\;\;=\;\;1\;\;-\;\;\rho$$

The heat balance equation taking the parabolic shape of the cooker into consideration is

(7) $$Q\;\;=\;\;E\;\;-\;\;\varepsilon_{1}\left(\sum \varphi_{i}J_{i}\right)$$

where *Q* is the heat absorbed, *E* is emission, ϕ is the shape factor, and *J* is the radiosity of the surfaces.

The energy efficiency is given as

(8) $$\eta \;\;=\;\;1\;\;+\;\;\frac{1}{3}{\left(\frac{T_{0}}{T}\right)}^{4}\;\;-\;\;\frac{4}{3}\frac{T_{0}}{T}$$

Considering the surface and emissivity of the cooker the modified energy efficiency of the cooker is given as

(9) $$\eta_{A}\;\;=\;\;100\;\;\times \;\;\frac{Q\left(1\;\;-\;\;\frac{T_{0}}{T}\right)}{I\varphi }$$

The energy loss of the system is calculated from the given formula

(10) $$\xi \;\;=\;\;100\frac{B}{I\varphi }$$

where *B* is the exergy loss.

The exergy loss during heat transferring from the cooking pot to the heating material is given by

(11) $$B_{1}\;\;=\;\;Q\left(\frac{T_{\mathrm{ft}}\;\;-\;\;T_{\mathrm{amb}}}{T_{\mathrm{ft}}}\;\;-\;\;\frac{T_{\mathrm{it}}\;\;-\;\;T_{\mathrm{amb}}}{T_{\mathrm{it}}}\right)$$

Heat loss due to convective heat transfer from surface to ambient is

(12) $$B_{2}\;\;=\;\;Q\left(\frac{T_{\mathrm{ft}}\;\;-\;\;T_{\mathrm{amb}}}{T_{\mathrm{ft}}}\right)$$

The total heat loss is given by

(13) $$B\;\;=\;\;\sum \phi_{i}Q_{i}\;\;-\;\;\sum Q_{i}\left(1\;\;-\;\;\frac{T_{\mathrm{amb}}}{T_{\mathrm{ft}}}\right)$$

## 4 Performance Using Normal Pot

At first an ordinary cooking vessel is used and its performance is studied. Water weighing 1 kg is heated and the values of water temperature are taken at an interval of 15 min using a thermometer. The performance is presented in **Table** **1**.

**Table 1:** Performance of DSC (parabolic thin plate type) with ordinary cooking pot (1 kg mass of water)

Time of day [IST]	Output fluid (water) temperature [K]	Ambient temperature [K]	Solar luminous intensity [lx] ×100	Solar radiation (*I*) [W m^−2^]	Input [W]		Output [W]		Efficiency [%]	
					Energy	Exergy	Energy	Exergy	Energy	Exergy
12:15	306.15	304.15	375.00	192.73	321.85	304.37	29.40	29.37	9.13	9.65
12:30	311.15	304.15	360.00	185.02	308.98	292.20	21.00	20.98	6.80	7.18
12:45	316.15	304.15	335.00	172.17	287.52	271.91	21.00	20.98	7.30	7.71
13:00	319.15	303.15	321.00	164.97	275.51	260.59	12.60	12.59	4.57	4.83
13:15	320.15	303.15	313.00	160.86	268.64	254.10	4.20	4.20	1.56	1.65
13:30	322.15	303.15	301.00	154.70	258.34	244.36	8.40	8.39	3.25	3.43
13:45	323.15	303.15	283.00	145.44	242.89	229.74	4.20	4.20	1.73	1.83
14:00	325.15	303.15	268.00	137.74	230.02	217.57	8.40	8.39	3.65	3.86
14:15	326.15	303.15	261.00	134.14	224.01	211.88	4.20	4.20	1.87	1.98
14:30	329.15	303.15	247.00	126.94	211.99	200.52	12.60	12.59	5.94	6.28
14:45	331.15	302.15	234.00	120.26	200.84	190.00	8.40	8.39	4.18	4.42
15:00	333.15	302.15	217.00	111.52	186.25	176.20	8.40	8.39	4.51	4.76
15:15	334.15	303.15	203.00	104.33	174.23	164.80	4.20	4.20	2.41	2.55

It can be observed that the total energy input to the solar cooker is 3525.38 W and the total output energy is 146.84 W, thus yielding an average efficiency of 4.06%. The total exergy input to the system is 3337.28 W and the total output exergy is 147 W, giving an efficiency of 4.29%. The peak energy efficiency obtained is 9.13% and it depends on the initial and final temperature, as the temperature of water increases the energy efficiency tends to decrease. Water is heated from 26 to 61 °C and the time taken is 195 min. **Table** **2** depicts the performance of the solar cooker for normal utensil. It can be observed that to heat 1 kg of water 195 min was needed. This is a substantial amount of time and there is a need to decrease this time. Solar cooker is clean and cheap source of cooking medium but suffers from the problem of higher cooking time, thus decreasing the overall effectiveness of the cooker. There is a need to decrease this cooking time and increase the overall effectiveness of cooking by using cost effective means. The second part of the experiment is done using the same but with activated carbon coating.

**Table 2:** Performance of DSC (parabolic thin plate type 2) with aluminium black coated cooking pot (1 kg mass of water)

Time of day [IST]	Output fluid (water) temperature [K]	Ambient temperature [K]	Solar luminous intensity [lx] x100	Solar radiation (*I*) [W m^−2^]	Input [W]		Output [W]		Efficiency [%]	
					Energy	Exergy	Energy	Exergy	Energy	Exergy
12:15	306.15	304.15	375.00	192.73	321.85	304.37	54.60	54.54	16.96	17.92
12:30	316.15	304.15	360.00	185.02	308.98	292.20	42.00	41.89	13.59	14.34
12:45	331.15	304.15	335.00	172.17	287.52	271.91	63.00	62.83	21.91	23.11
13:00	334.15	303.15	321.00	164.97	275.51	260.59	12.60	12.41	4.57	4.76
13:15	333.15	303.15	313.00	160.86	268.64	254.10	−4.20	−4.38	−1.56	−1.72
13:30	333.15	303.15	301.00	154.70	258.34	244.36	0.00	−0.18	0.00	−0.07
13:45	332.15	303.15	283.00	145.44	242.89	229.74	−4.20	−4.38	−1.73	−1.91
14:00	331.15	303.15	268.00	137.74	230.02	217.57	−4.20	−4.37	−1.83	−2.01
14:15	329.15	303.15	261.00	134.14	224.01	211.88	−8.40	−8.56	−3.75	−4.04
14:30	327.15	303.15	247.00	126.94	211.99	200.52	−8.40	−8.56	−3.96	−4.27
14:45	327.15	302.15	234.00	120.26	200.84	190.00	0.00	−0.15	0.00	−0.08
15:00	325.15	302.15	217.00	111.52	186.25	176.20	−8.40	−8.55	−4.51	−4.85
15:15	323.15	303.15	203.00	104.33	174.23	164.80	−8.40	−8.54	−4.82	−5.18

## 5 Performance Using Aluminium Charcoal Coated Pot

The Same experiment is performed using aluminium activated carbon coated pot. Activated carbon is black carbon and ash residue. It is obtained by burning wood or plants in the absence of oxygen. In India people mostly use firewood for cooking, so activated carbon is available and is cheap. Coating the aluminium pot with activated carbon is cost effective as it is abundantly available. One kilogram of water is heated and the performance is presented in Table 2.

In this case the total energy input to the solar cooker is 3528.381 W and the total output energy is 218.4 W, thus yielding an average efficiency of 5.65%. The total exergy input to the system is 3337.281 W and the total output exergy is 219.335 W, giving an efficiency of 6.01%. The peak energy efficiency obtained is 21.91% and it depends on the initial and final temperature, as the temperature of water increases the energy efficiency tends to decrease. Water is heated from 26 to 61 °C and the time taken was 60 min. It can be seen that by using aluminium activated carbon coated pot took 60 min to heat the water to 61 °C. From Table 2 it is observed that the output energy and exergy are becoming negative at certain times, this due to the fact that the temperature gradient is becoming negative.

## 6 Result Analysis

The performance of parabolic thin plate type solar pot with ordinary aluminium pot and activated carbon coated aluminium pot are tabulated in Tables 1 and 2, respectively. The ambient temperature of air is nearly constant and the solar luminous intensity varied from 203 000 to 393 000 lx.

**Figure 3:**
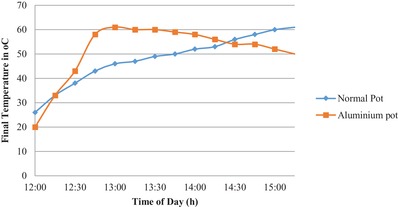
Variation of final water temperature with time.

**Figure 4:**
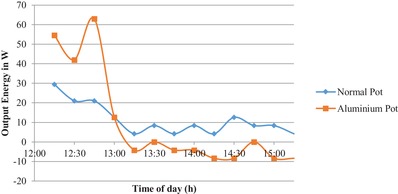
Variation of output energy with time.

**Figure 5:**
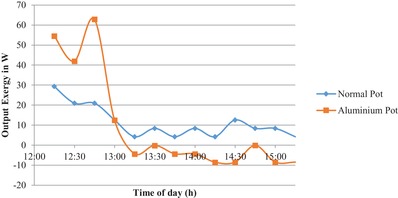
Variation of output energy with time.

It can be observed that by using activated carbon coated aluminium pot the maximum temperature of 62 °C is reached after 60 min whereas for normal aluminium pot the maximum temperature is reached after 195 min. There is a difference of 120 min. This denotes a significant reduction in cooking time, thus increasing the effectives of cooking. From **Figure** **3** it can be observed that the temperature gradient by using activated carbon coated aluminium pot is steep. This denotes that the rise in temperature of the coated pot is higher than the normal pot. This gives a clear indication that the cooking time will decrease considerably by using the coated aluminium pot. In **Figures** **4** and **5** the output energy and the output exergy is plotted respectively. It is observed that the output energy and the exergy of the activated carbon coated aluminium pot are higher than that of the normal pot. The maximum energy and exergy of the activated carbon coated aluminium pot is 63 W whereas the maximum output energy and output exergy of the normal pot is 9 W.

By using the activated carbon coating the output energy is increased by 54 W. This is a substantial increase in the energy output of the cooker which increases the cooking effectiveness and decrees the cooking time. In the first hour of the experiment the slope of temperature rise was steeper from 12:00 p.m. to 13:30 p.m. and after the slope became flatter, it is because the heat conduction is a function of both temperature difference and input solar radiation. From Figures 4 and 5 it can be observed that maximum output energy and exergy is obtained at 12:45 p.m. when the temperature difference is maximum.

**Figure 6:**
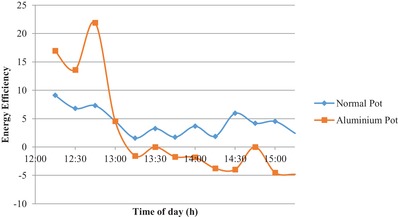
Efficiency of the pots with time.

**Figure** **6** shows the energy and exergy efficiency of the system. The average energy efficiency for normal aluminium pot was found to be 4.06% and for activated carbon coated aluminium pot it was 5.65%. The maximum energy efficiency for the activated carbon coated aluminium pot is 22% and is obtained at 12:45 pm. The maximum energy efficiency of the normal pot is 9%. There is a significant increase in the efficiency of solar cooker using the activated carbon coating.

Findings from the experiment:

- Using activated carbon coated pot for cooking to reach maximum temperature it took less amount of time.
- The maximum temperature is reached after 60 min for activated carbon coated pot and for the normal pot it took 195 min.
- The energy output of the solar cooker increased 32 W by using activated carbon coated pot in comparison to the energy output when normal pot is used.
- The exergy output for the coated pot increases.
- The energy efficiency of the solar cooker increased by using activated carbon coated aluminium pot.
- The average energy for the coated pot is 5.65% and for the normal pot is 4.06%.

The effectiveness of the solar cooker is increased considerably by using activated carbon coating on the cooking pot. This provides a cheap and effective way to reduce cooking time in solar cooker.

## 7 Conclusion

The energy efficiency and exergy efficiency of a thin plate type solar cooker was determined experimentally using two different type of pots. The activated carbon coated aluminium pot took 60 min to reach the highest temperature while the normal aluminium pot took 195 min to reach the same temperature, which is more than three times the time taken by activated carbon coated aluminium pot. The solar cooker performance of activated carbon coated aluminium pot in terms of efficiency was high and had a lower cooking time. The efficiency of activated carbon coated aluminium pot is 1.59% more than the normal aluminium pot. Using activated carbon coated cooking pot in solar cooker provides a cost effective solution and increases the effectiveness of the cooker. Considering the economic condition this method will provide a cheap, clean and faster cooking.

## Conflict of Interest

The authors declare no conflict of interest.
